# Assessing ethnic inequalities in diagnostic intervals of breast cancer among patients presenting symptoms to general practitioners in England

**DOI:** 10.1038/s41598-026-36070-8

**Published:** 2026-01-28

**Authors:** Tanimola Martins, Deepthi Lavu, William Hamilton, Gary Abel, Richard D. Neal

**Affiliations:** University of Exeter Collaboration for Academic Primary Care (APEx), University of Exeter Medical School, Exeter, EX1 2LU UK

**Keywords:** Cancer, Diseases, Health care, Medical research, Oncology

## Abstract

This retrospective-cohort study examined ethnic differences in breast cancer diagnostic intervals among women aged aged ≥40 years who were diagnosed in England between 2017 and 2021. We analysed primary care-linked hospital data, with diagnostic interval divided into primary care interval (period between first presentation and referral), referral interval (period between referral and first specialist appointment), and secondary care interval (period between first specialist appointment and diagnosis). Across the cohort, the median diagnostic interval was 24 days and was longer for women presenting with non-lump symptoms than for those with a breast lump (ATR = 1.56, 95%CI: 1.31–1.86). Black patients had longer diagnostic interval than White patients regardless of symptom type: [ATR = 1.38, 95%CI:1.11–1.71]. The median primary care interval was 1 day. It was longer for women with non-lump symptoms than those with a breast lump [ATR = 1.49, 95%CI:1.16–1.92], but shorter for Asian patients, especially those with a breast lump, compared to White patients [ATR = 0.89, 95%CI: 0.84–0.96]. The median referral interval was 11 days, with no evidence of variation by symptom type or ethnicity. The secondary care interval was also 11 days and was longer for patients with non-lump symptoms [ATR = 1.41, 95%CI:1.14–1.74] and for Black patients, particularly those with a breast lump, compared to White patients [ATR = 1.66, 95%CI:1.21–2.28]. Our findings highlight the need for targeted interventions to address ethnic disparities in breast cancer outcomes, including both lump and non-lump symptom presentations. Such interventions should prioritise optimising the entire diagnostic pathway—from initial presentation through to diagnosis—rather than concentrating solely on primary care, ensuring timely and equitable access across all healthcare settings.

**Supplementary Information:**

The online version contains supplementary material available at 10.1038/s41598-026-36070-8.

## Introduction

Breast cancer remains a leading cause of cancer-related deaths among women worldwide. In the UK, approximately 55,500 new cases and over 11,000 deaths from breast cancer occur annually^1,2^. The availability of effective screening, early diagnosis initiatives, and treatment options have generally improved both clinical and patient outcomes, but these outcomes vary considerably depending on the stage at diagnosis^3^. Outcomes also differ by sociodemographic factors such as age, socio-economic status, and in particular, ethnicity. This is reflected by the fact that, despite a lower incidence of breast cancer^4^, UK Asian and Black women are more likely to be diagnosed at a later stage, have poorer survival rates, and report less positive healthcare experiences compared to their British White counterparts^5–7^. These disparities have been linked, in part, to patient-related factors, including lower awareness of early warning signs, reduced participation in mammography screening, and delays in medical help-seeking—issues more prevalent among minority ethnic groups^8–12^.

However, healthcare-related factors may also contribute to inequalities in breast cancer outcomes, although only a few UK studies have specifically explored this association. A recent analysis of English cancer registry data found that, on average, Asian and Black women presenting with breast cancer symptoms in primary care are more likely than White women to experience a longer diagnostic interval: the period between initial symptomatic presentation and cancer diagnosis^13^. A follow-up study, using the same data, attributed these differences mainly to the length of time spent in primary care before secondary care referral^14^. However, both studies focused on patients diagnosed between 2006 and 2016, and are therefore somewhat dated. They also did not account for the role of presenting symptoms or the impact of COVID-19 and associated measures on the timeliness of diagnosis.

The type of symptoms reported during primary care consultation is particularly critical as this may contribute to diagnostic interval^15^. On average, women who present with a breast lump are more likely to receive a faster referral and diagnosis than those presenting with non-lump symptoms (e.g., breast pain or nipple changes)^15^. However, a recent analysis of primary care records in England indicated that around 10% of Black women had breast pain as the first (and only) symptom recorded before diagnosis^16^. Breast pain is common and less often associated with breast cancer^17^ so not considered a criterion for urgent specialist referral under current clinical guidelines. No UK studies have specifically investigated the role of symptom types on ethnic differences in breast cancer diagnostic interval.

In this study, we used linked primary and secondary care data to investigate ethnic differences in total, primary care, referral and secondary care intervals of breast cancer diagnosis. We performed secondary analyses to examine the role of symptom type (lump or non-lump) and COVID-19 lockdown on each interval of diagnosis.

## Methods

### Design and databases

We conducted a retrospective cohort study of English cancer patients using data from the Clinical Practice Research-Datalink (CPRD-Aurum) linked to Hospital Episode Statistics (HES). The CPRD is the world’s largest primary care database, with linkages to other health and area-based datasets used in this study^18–21^. The CPRD-Aurum used in this study (May 2022 build), comprised routinely gathered data from 1,491 consenting English practices, with over 38 million patients eligible for linkage to other health care databases including HES and Index of Multiple Deprivation (IMD)^20^. The dataset is representative of the UK population in terms of geography, deprivation, age, gender, and ethnicity^18,19,22^. It included coded and anonymised data on patients’ medical history (including reported breast cancer symptoms), investigations, diagnoses, prescriptions, referral, and demographics (age, gender, and ethnicity)^18,19,22^. HES data—Admitted patient care and Outpatient elective—contains clinical and administrative data on all National Health Services (NHS) hospital admissions and outpatient appointments in England^18,19,22^.

### Cancer sites and participants

Eligible patients were adult females, aged ≥ 40years, with a recorded breast cancer diagnosis in the CPRD between January 2017 and August 2021 who also had a recorded breast cancer symptom in the year before diagnosis recording.

### Participants’ ethnicity

Ethnicity information was initially sourced from the CPRD and supplemented with HES records if missing in the CPRD, as previously described^23,24^. We defined ethnicity using the UK’s census groupings, which includes the following: White (White British, White Irish, Any other White); Asian (Indian, Pakistani, Bangladeshi, Chinese, Other Asian); Black (Black Caribbean, Black African, Other Black); Mixed(White & Black Caribbean, White & Black African, White & Asian, Any other mixed); and Other ethnic group. For patients with multiple ethnicity codes, we used the most frequently recorded codes or in their absence, the most recently recorded code following established methods^23,24^. Those with missing or unknown ethnicity records in the CPRD or HES (< 2%) were coded as Other for analysis.

### Recorded breast cancer symptoms

As in previous studies^13,25,26^, we identified possible breast cancer symptoms from the CPRD, based on the National Institute for Health and Care Excellence (NICE) guidance^27^. These symptoms included breast lump, and non-lump symptoms – namely breast pain, nipple retraction, nipple discharge, axillary lymphadenopathy and breast skin changes (including puckering, dimpling and rash formation). The index symptom was defined as the first symptom recorded in the year before diagnosis, as in previous studies^13,26^.

### Specialist referrals

Data on cancer specialist referral were available in CPRD and HES. We matched CPRD records of patients with breast cancer symptoms to relevant oncological specialties in HES, specifically “General Surgery” and “Breast Surgery services”. Only patients with a recorded appointment date with these specialists, and a referral source of “General Practitioner” or “General Practitioner with an Extended Role” were retained for analysis. For patients with multiple referral records prior to diagnosis, the referral date was defined as the earliest referral request in HES after the index symptom date, and the specialist appointment date as the earliest appointment following that referral. The final dataset included patients with valid dates for the index symptom, referral request, and specialist appointment.

### Defining diagnostic intervals:

We identified four distinct intervals within the diagnostic pathway^28^: a) the primary care interval (PI)—the period between the dates of first symptomatic presentation in primary care and referral to secondary care; b) referral interval (RI)—the period between the dates of referral and first appointment in secondary care; c) secondary care interval (SI)—the period between the dates of first secondary care appointment and eventual diagnosis; and d) total interval [diagnostic interval hereafter (DI)]- the period between first primary care presentation and diagnosis.

### Other variables

Information on patient age, level of deprivation and co-morbidities were identified from the CPRD. The date of birth required assigning a nominal birthday of 1st July to each patient, as only the year of birth was available. Patient socioeconomic deprivation used quintiles of the 2019 IMD—a composite measure of deprivation for small areas in England – based on patient’s residence postcode^22^. Co-morbidities recorded for each patient before cancer diagnosis (identified from the CPRD) were used to derive Cambridge Multimorbidity Scores (CMS), based on the General-outcome weighting described in Payne et al^29,30^. Four morbidity burden groups were defined with one group containing those with no included morbidities, and the rest categorised according to tertiles of the CMS scores.

### Statistical analysis

We used Accelerated Failure Time (AFT) models to analyse the association between ethnicity and diagnostic intervals. AFT models provide time ratios, where a ratio > 1 indicates a longer interval and < 1 indicates a shorter interval in an ethnic minority group compared to the White group. Both crude and adjusted time ratios were reported. We fitted three separate models. Model 1 was an unadjusted analysis examining ethnic differences in diagnostic intervals, using robust standard errors to account for clustering within practices. Model 2 built on Model 1 by adjusting for potential confounders, including age, deprivation, comorbidity, and year of diagnosis. The year of diagnosis was included as a binary variable, distinguishing the period before COVID-19 lockdown (January 2017—March 2020) from the lockdown period (March 2020—August 2021). Model 3 further extended Model 2 by incorporating the mediating effect of symptom type (breast lump vs. non-lump symptoms) on the diagnostic interval. Additionally, we performed a supplementary analysis to explore two-way interactions between ethnicity and symptom type, following methods from previous studies^23^. Briefly, we included an interaction term (ethnicity#symptom_type) in Model 3 and assessed its significance using likelihood ratio tests (*p* < 0.05). Where the results indicate a significant interaction between these variables, we performed stratified analyses by symptom type. Finally, we performed sensitivity analyses excluding cases diagnosed from March 2020 onward to account for the potential effects COVID-19 lockdown on diagnostic intervals. All analyses were performed in Stata v18, with the results reported in accordance with the STROBE guidelines for cohort studies^31^.

## Results

### Cohort characteristics

Our cohort comprised 38,537 patients. We excluded patients without recorded breast cancer symptoms [n = 22,835], those referred from non-GP sources [n = 805], those missing valid specialist referral dates [n = 4,424] or missing a record of attendance at a breast specialist [n = 1,774], and those under 40 years old at diagnosis [n = 77]. Table 1 outlines participants’ demographic characteristics. The majority (85%) were diagnosed before the COVID-19 lockdown [6,290 (85%)] and were predominantly White patients [7,390 (86%)], with smaller proportions of Mixed[464 (5%)], Asian [366 (4%)], Black [228 (3%)], and Other ethnic group [174 (2%)]. At diagnosis, Asian and Black patients were, on average, younger and had a greater percentage living in the most deprived areas compared with White patients. The percentage of patients with any recorded morbidities was highest in the White group.

**Table 1:** Participant characteristics.

Variable	Sub-categories	White	Black	Asian	Mixed	Other	All
Age (Years)	Median (IQR)	64 (51–78)	54 (48–63)	52 (46–65)	60 (49–74)	60 (49–71)	63 (50–77)
	40–49	1648 (22.3)	83 (36.4)	152 (41.5)	134 (28.9)	52 (29.9)	2069 (24.0)
	50–59	1532 (20.7)	75 (32.9)	84 (22.9)	102 (21.9)	36 (20.7)	1829 (21.2)
	60–69	1149 (15.6)	32 (14.0)	62 (16.9)	76 (16.4)	41 (23.6)	1360 (15.8)
	70 >	3061 (41.4)	38 (16.7)	68 (18.6)	152 (32.8)	45 (25.9)	3364 (39.0)
Deprivation n (%) *	Least deprived—1	1937 (26.2)	5 (2.19)	57 (15.6)	96 (20.7)	43 (24.7)	2138 (24.8)
	2	1818 (24.6)	18 (7.89)	56 (15.3)	128 (27.6)	58 (33.3)	2078 (24.1)
	3	1370 (18.5)	36 (14.9)	77 (21.0)	86 (19.0)	27 (15.5)	1594 (18.5)
	4	1179 (15.9)	91 (39.9)	103 (28.1)	94 (20.3)	25 (14.4)	1492 (17.3)
	Most deprived -5	1081 (14.6)	80 (35.1)	73 (19.9)	60 (12.3)	20 (11.5)	1314 (15.2)
Morbidity, n (%)	None	1291 (17.5)	46 (20.2)	90 (24.6)	96 (20.7)	57 (32.8)	1580 (18.3)
	1	2482 (33.6)	88 (38.6)	120 (32.8)	172 (37.1)	64 (36.8)	2926 (33.9)
	2	1888 (26.0)	52 (23.0)	90 (24.6)	103 (22.0)	40 (22.9)	2173 (25.0)
	3 (most score)	1729 (23.4)	42 (18.4)	66 (18.0)	93 (20.0)	13 (7.00)	1943 (22.5)
Year of diagnosis n(%)	Pre- lockdown	6290 (85.1)	199 (87.3)	311 (84.9)	381 (82.1)	148 (85.1)	7329 (85.0)
All	Total	7,390 (85.7)	228 (2.64)	366 (4.24)	464 (5.38)	174 (2.02)	8,622 (100)

Missing deprivation record [n = 6, White(n = 5) and Other (n = 6).

### Index symptoms

Breast lump was the most common index symptom, recorded in 92% of White, 91% of Mixed, 90% of Asian and Black, and 94% of Other ethnic group (Supplementary File S1). Breast pain was the second most commonly recorded index symptom, with varying proportions by ethnicity: 6% among White and Mixed, 8% among Black, 9% among Asian, and 4% among those in Other ethnic group.

### Diagnostic intervals by symptom type

The median, 25^th^, 75^th^, and 95^th^ percentiles of each interval of diagnosis by recorded symptom type are illustrated in Supplementary File S2, while the results of crude and adjusted analyses are available in Supplementary File S3. After adjusting for age, deprivation, comorbidities, and year of diagnosis, we found that patients with recorded non-lump symptoms had a longer DI compared to patients with a recorded breast lump [adjusted time ratio (ATR) = 1.56, 95% confidence interval (CI):1.31–1.86]. Similarly, patients with recorded non-lump symptoms had longer PI [ATR = 1.49, 95% CI:1.16–1.92] and SI [ATR = 1.41, 95% CI:1.14–1.74] compared to patients with a recorded breast lump. However, no difference was observed in RI based on symptom type.

### Ethnic differences in diagnostic interval (DI)

**Fig. 1:**
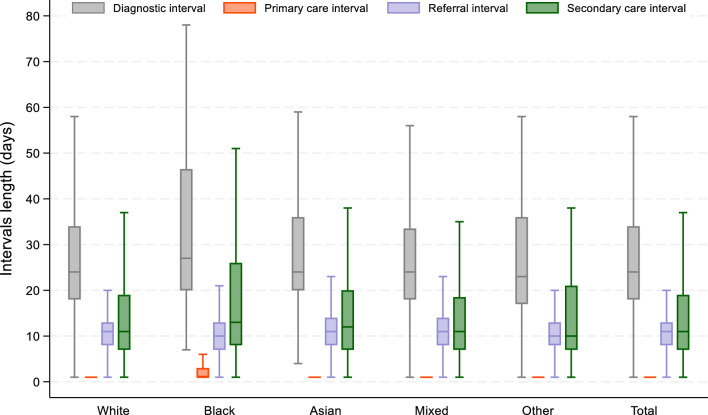
Boxplots showing ethnic differences in diagnostic intervals.

Figure 1 comprises boxplots of diagnostic intervals by ethnicity, with details on the medians and associated 25^th^, 75^th^, and 95^th^ percentiles provided in Supplementary File S2. Across all ethnic groups, the median DI was 24 days [interquartile range (IQR):18–34], ranging from 23days (36–99) among patients from the Other ethnic group to 27days (47–171) in Black patients. In adjusted analyses (Table 2, Model 2), the DI was typically 39% longer for Black patients compared with White patients [ATR = 1.39,95%CI:1.13–1.71]. Further adjustment for symptom type (in Model 3) had little impact on this association [ATR = 1.38, 95%CI: 1.11–1.71], and no significant difference in DI was observed between Asian, Mixed, and Other groups compared with the White group.

**Table 2:** Association between ethnicity and diagnostic intervals.

Diagnostic interval										
Ethnicity	Total	Model 1			Model 2			Model 3		
		CTR	95% CI	P-value	ATR	95% CI	P-value	ATR	95% CI	P-value
White	7,390									
Black	228	1.36	1.12–1.64	0.002	1.39	1.13–1.71	0.002	1.38	1.11–1.71	0.003
Asian	366	1.01	0.89–1.12	0.92	0.97	0.85–1.11	0.70	0.97	0.85–1.11	0.69
Mixed	464	1.01	0.89–1.14	0.86	0.99	0.86–1.13	0.85	0.99	0.87–1.13	0.86
Other	174	1.02	0.81–1.29	0.87	0.93	0.72–1.20	0.58	0.93	0.73–1.19	0.57
Total	8,622									
Primary care interval										
White	7,390									
Black	228	1.24	0.97–1.59	0.08	1.21	0.93–1.56	0.16	1.19	0.91–1.54	0.20
Asian	366	0.97	0.89–1.05	0.43	0.88	0.81–0.96	0.004	0.88	0.81–0.96	0.004
Mixed	464	0.97	0.89–1.06	0.51	0.98	0.88–1.09	0.66	0.96	0.87–1.08	0.52
Other	174	0.93	0.79–1.07	0.30	0.88	0.75–1.05	0.16	0.88	0.73–1.06	0.19
Total	8,622									
Referral interval										
White	7,390									
Black	228	0.99	0.85–1.15	0.94	0.98	0.83–1.16	0.80	0.98	0.83–1.16	0.80
Asian	366	0.98	0.89–1.07	0.62	0.98	0.88–1.09	0.69	0.98	0.88–1.09	0.69
Mixed	464	1.00	0.89–1.11	0.98	0.99	0.87–1.12	0.84	0.99	0.87–1.11	0.81
Other	174	0.87	0.72–1.06	0.16	0.95	0.76–1.19	0.66	0.95	0.76–1.19	0.67
Total	8,622									
Secondary care interval										
White	7,390									
Black	228	1.44	1.08–1.92	0.01	1.46	1.07–2.00	0.02	1.45	1.05–2.01	0.02
Asian	366	1.05	0.88–1.26	0.57	1.05	0.83–1.31	0.69	1.05	0.84–1.31	0.69
Mixed	464	1.00	0.81–1.23	1.00	0.95	0.75–1.20	0.66	0.95	0.75–1.21	0.68
Other	174	1.27	0.85–1.88	0.24	1.01	0.66–1.54	0.97	1.02	0.68–1.53	0.94
Total	8,622									

Model 1: Unadjusted; Model 2: Adjusted for age, deprivation, comorbidity, and year of diagnosis (pre- vs. during COVID-19 lockdown); Model 3: Model 2 plus symptom type as mediator (breast lump vs. non-lump).

### Ethnic differences in primary care interval (PI)

The median PI was 1day [IQR: 1–1], with little evidence of a difference by ethnicity (Fig. 1, Supplementary File S2). Both multivariable models showed that the PI was shorter for Asian patients compared to White patients[ATR = 0.88, 95% CI: 0.81–0.96] (Table 2). There was no evidence of a difference in PI between Black, Mixed, Other and White patients.

### Ethnic differences in referral interval (RI)

The median RI was 11days [IQR: 7–13], with no evidence of ethnic differences identified in either crude or adjusted analyses (Table 2).

### Ethnic differences in secondary care interval (SI)

The median SI was 11days [IQR: 7–19], and it was longer for Black patients compared with White patients. In adjusted analyses (Table 2, Model 2), the SI was 46% longer for Black patients compared with White patients [ATR = 1.46, 95%CI:1.07–2.00]. Further adjustment for symptom type (Model 3) had little effect on this association [ATR = 1.45, 95%CI:1.05–2.01]. There was no evidence of a difference in SI between Asian, Mixed, Other and White patients.

### Supplementary analyses

The test of interaction analyses found no evidence that the effect of ethnicity on the DI or RI varied by symptom type. However, there was some evidence (*p* = 0.05) that ethnic differences in the PI varied by symptom type. Stratified analyses by symptom type indicated that the PI was shorter for Asian patients presenting with a breast lump (ATR = 0.89, 95%CI: 0.84–0.96), a difference not observed among those with non-lump symptoms or in other ethnic groups (Table 3). In contrast, there was strong evidence (*p* < 0.0001) that ethnic differences in the SI varied by symptom type. Stratified analyses showed that, among patients with a breast lump, the SI was 66% longer for Black patients compared to White patients (ATR = 1.66, 95%CI:1.21–2.28); but no difference was observed for patients with non-lump symptoms (Table 3).

**Table 3:** Association between ethnicity and diagnostic intervals by symptom type.

Primary care interval										
	Total	Lump					Non-lump			
		CTR	ATR	95% CI	P-value	Total	CTR	ATR	95% CI	P-value
White	6827	-				563				
Black	206	1.21	1.21	0.95–1.55	0.13	22	1.07	0.92	0.25–3.49	0.91
Asian	331	0.95	0.89	0.84–0.96	0.002	35	2.49	1.50	0.35–6.39	0.36
Mixed	422	0.97	0.97	0.89–1.07	0.54	42	0.72	0.86	0.27–2.71	0.79
Other	163	0.97	0.92	0.78–1.09	0.32	11	0.42	0.63	0.27–1.48	0.29
Total	7949		673							
Secondary care interval										
White	6827	-				563				
Black	206	1.64	1.66	1.21–2.28	0.002	22	0.48	0.51	0.22–1.19	0.12
Asian	331	0.99	0.98	0.78–1.23	0.86	35	2.12	2.04	0.79–5.29	0.14
Mixed	422	0.98	0.95	0.75–1.20	0.68	42	1.18	1.08	0.27–4.30	0.92
Other	163	1.14	0.96	0.62–1.47	0.83	11	2.85	1.48	0.82–2.67	0.19
Total	7949					673				

ATR—Adjusted for age, deprivation, comorbidity, and year of diagnosis (pre- vs. during COVID-19 lockdown).

Sensitivity analyses showed that excluding cases diagnosed from March 2020 onward did not materially change the results (Supplementary File S4). Diagnostic intervals were consistent across models, indicating that COVID-related disruptions had minimal impact on our estimates.

## Discussion

The median DI (24days) in our 2017–2021 cohort is longer than the 13-day average reported in a study of patient diagnosed between 2006 and 2016.(13) However, that study included patients diagnosed via non-primary care routes (e.g., accident and emergency and other hospital routes), whereas our study focused exclusively on patients diagnosed via the primary care route. While our estimated diagnostic interval falls well within the 28-day standard outlined in the current NHS guidelines for Faster Diagnostic pathways from a GP’s referral, it does not tell the whole story. For instance, consistent with findings from previous studies^15^, patients with recorded non-lump symptoms in our study had a longer DI than those with a breast lump. The median (75^th^–95^th^ percentile) DI for patients with non-lump symptoms in our cohort was 32 days (70–280) compared to 23 days (33–84) for those with a breast lump (Supplementary File S2). The 75^th^ and 95^th^ percentiles are particularly concerning, especially for women with non-lump symptoms, who may be overrepresented among those diagnosed with advanced-stage breast cancer. It is possible that these patients were not referred via the fast-track referral route, as non-lump symptoms, such as breast pain, are not considered a referral criterion under NICE guidelines^27^. Further research is necessary to fully understand the impact of non-lump symptoms on breast cancer presentation, referral, specialist investigations, and associated outcomes.

Contrary to our hypothesis, a major fraction of the interval of diagnosis could be attributed to prolonged SI. For instance, for patients with non-lump symptoms, the median SI was 15days (75th—95th percentiles: 34–207) compared to a median PI of just 1day (4–104). A similar pattern was observed for those with a breast lump, where the median PI was 1day (1–8) versus a median SI of 11days (18–61). The 75^th^ and 95^th^ percentile estimates are particularly important considering that the likelihood of a cancer diagnosis—and the associated psychological distress—typically increase upon referral to secondary care, with the SI generally assumed to be shorter than the primary care interval^32,33^. To our knowledge, no UK studies have specifically explored the factors contributing to SI delays in breast cancer, highlighting a potential area for future research.

The average DI was longest in Black patients compared to White patients, even after accounting for potential confounding factors, including the effect of symptom type and COVID-19 lockdown. Upon further analysis, we found that this difference was driven largely by prolonged SI in Black women, especially in those with a breast lump. This is unexpected and rather concerning, as a lump is a well-recognised indicator of breast cancer, warranting a faster diagnostic pathway^27^. These findings suggest, that while initial primary care presentation and referral might be similar, Black women experience undue delay in secondary care, the determinants of which warrants further investigation. Psycho-social factors such as fear of cancer, its treatment, and concerns about abandonment by partner have all been linked to delayed help-seeking in primary care^34^. It is possible these contribute to prolonged SI in Black women. On the other hand, health-system factors (e.g., distance to hospital, workload, and hospital communication/information delivery systems), have all been linked to diagnostic delays in South-East Asia^35^, but no UK studies have explored the determinants of ethnic differences in SI of breast cancer diagnosis. If patient-related factors contribute to prolonged SI of diagnosis in Black women, then interventions like One-Stop Breast Clinics in the UK^36^ may have limited value for women from this group, and other marginalised groups. Therefore, an in-depth exploration of the potential determinants of secondary care delays, particularly across sociodemographic groups, is essential.

Asian women, especially those with a breast lump had a shorter PI compared to White women. However, we found no difference in RI or SI between the two groups. Additionally, we did not find any difference between the Mixed and Other groups compared with the White group across any of the diagnostic intervals.

## Strengths and limitations

Our cohort included patients diagnosed between 2017 and 2021 and assessed the impact of symptom type and COVID-19 on the diagnostic interval for breast cancer. Cases were identified from the CPRD-linked hospital data, the world’s largest primary-care database, known for its high-quality data. However, around 9% of breast cancers in the national cancer registry are deemed to be missing from the CPRD, with diagnosis dates recorded up to seven days later in the CPRD than in the registry data^37^. Our cohort was limited to patients aged 40 years or older, acknowledging that around 4% of breast cancers occur in younger individuals^38^. Future research should include younger patients, particularly Asian and Black women, who are more likely to be diagnosed at earlier ages. We restricted our analysis to patients referred by the GP, with valid dates for index symptoms, referral requests, and specialist appointments. This led to the exclusion of about three-quarters of cases (n = 29,915), 78% of whom had no recorded breast cancer symptoms and 21% were missing valid referral or specialist appointment dates. Around 30% of those without recorded symptoms were likely diagnosed through breast screening^23,39^, making them ineligible for our study, which focuses solely on symptomatic presentations. The proportion (21%) missing valid referral or specialist dates highlights the need for improved data capture within the NHS. Overall, while these exclusions may have introduced some bias by omitting patients who presented in other settings (e.g., emergency departments) or those with non-NICE qualifying symptoms in primary care, they ensure that our results accurately estimate diagnostic intervals involving the GP. The excluded patients were broadly comparable to those included (results not shown), suggesting minimal impact from these exclusions. For simplicity, we used combined ethnic categories, recognising that this approach obscures diversity within ethnic subgroups.

## Conclusion

This study adds to our understanding of the determinants of ethnic inequalities in breast cancer outcomes, emphasising the complex roles of recorded symptoms (also a proxy for symptoms reported) in the diagnostic pathways. While the overall DI in our study aligns with current Standard for Faster diagnoses, significant disparities were observed. A real concern lies with women with non-lump symptoms whose DI fall within the 75^th^ and 95^th^ percentiles (70–280 days), driven largely by prolonged SI, an area that has historically received limited attention in research. Such lengthy intervals are likely to negatively impact prognosis, necessitating more aggressive treatment and contributing to poorer care experiences.

Black women experienced significantly longer DI than White women, even when the presenting feature was a breast lump. Again, much of this was driven by prolonged SI rather than PI. Conversely, Asian women tended to have shorter PI, with no significant differences observed in RI or SI compared to White women. Overall, these findings highlight the need for effective interventions, particularly for women with non-lump symptoms and in secondary care, to address ethnic disparities in breast cancer outcomes. Achieving this requires improvements in clinical data capture and a deeper exploration of the socio-cultural and systemic barriers affecting diagnostic pathways. Future research should focus on identifying the drivers of secondary care delays and evaluating innovative diagnostic approaches, such as One-Stop Breast Clinics, to reduce ethnic inequalities and improve breast cancer care and outcomes for all patients.

## Supplementary Information

Supplementary Information 1.

Supplementary Information 2.

## Data Availability

The data supporting the findings of this study are available from the Clinical Practice Research Datalink (CPRD) subject to licensing restrictions and are therefore not publicly available. Access to the data may be requested directly from CPRD; further information is available from the corresponding author.
