# Supplementary material for: Assessing ethnic inequalities in diagnostic intervals of breast cancer among patients presenting symptoms to general practitioners in England
Source: Sci Rep. 2026 Jan 28;16:6514. doi: 10.1038/s41598-026-36070-8 (PMC12909917; doi:10.1038/s41598-026-36070-8)
Supplement: Supplementary file 2 — Supplementary Information 2. [file 41598_2026_36070_MOESM2_ESM.docx]

**Supplementary Files**

**S1: Ethnic differences in recorded symptoms**

|  | **White (n=7390)** | **Black (n=228)** | **Asian (n=366)** | **Mixed (n=464)** | **Other (n=174)** | **All (N=8622)** |
| --- | --- | --- | --- | --- | --- | --- |
| Breast pain | 404 (5.47) | 18 (7.89) | 32 (8.74) | 29 (6.25) | 6 (3.45) | 489 (5.67) |
| Breast lump | 6827 (92.4) | 206 (90.4) | 331 (90.4) | 422 (90.9) | 163 (93.7) | 7949 (92.2) |
| Nipple discharge | 95 (1.29) | 3 (1.32) | 2 (0.55) | 9 (1.94) | 3 (1.72) | 112 (1.30) |
| Nipple retraction | 38 (0.51) | 1 (0.44) | 1 (0.27) | 2 (0.43) | 1 (0.57) | 43 (0.50) |
| Lymphadenopathy | 3 (0.04) | 0 (0) | 0 (0) | 0 (0) | 0 (0) | 3 (0.03) |
| Breast skin changes | 23 (0.31) | 0 (0) | 0 (0) | 2 (0.43) | 1 (0.57) | 26 (0.30) |

**S2: Diagnostic intervals (days) by ethnicity and index symptom type**

| **Intervals** | Ethnicity | **All patients** | | | | **Lump** | | | | **Non-lump** | | | |
| --- | --- | --- | --- | --- | --- | --- | --- | --- | --- | --- | --- | --- | --- |
|  |  | p25 | p50 | p75 | p95 | p25 | p50 | p75 | p95 | p25 | p50 | p75 | p95 |
| Diagnostic | White | 18 | 24 | 34 | 102 | 18 | 23 | 32 | 83 | 22 | 32 | 69 | 278 |
|  | Black | 20 | 27 | 46.5 | 171 | 20 | 27 | 43 | 77 | 15 | 35.5 | 117 | 332 |
|  | Asian | 20 | 24 | 36 | 113 | 19 | 24 | 34 | 87 | 24 | 34 | 90 | 292 |
|  | Mixed | 18 | 24 | 33.5 | 123 | 18 | 24 | 32 | 109 | 17 | 33.5 | 71 | 253 |
|  | Other | 17 | 23 | 36 | 99 | 16 | 22 | 35 | 81 | 24 | 41 | 67 | 329 |
|  | All | 18 | 24 | 34 | 105 | 18 | 23 | 33 | 84 | 22 | 32 | 70 | 280 |
|  | | | | | | | | | | | | | |
| Primary care | White | 1 | 1 | 1 | 10 | 1 | 1 | 1 | 8 | 1 | 1 | 5 | 103 |
|  | Black | 1 | 1 | 3 | 21 | 1 | 1 | 3 | 18 | 1 | 1 | 3 | 250 |
|  | Asian | 1 | 1 | 1 | 14 | 1 | 1 | 1 | 10 | 1 | 1 | 15 | 140 |
|  | Mixed | 1 | 1 | 1 | 12 | 1 | 1 | 1 | 9 | 1 | 1 | 3 | 49 |
|  | Other | 1 | 1 | 1 | 13 | 1 | 1 | 1 | 13 | 1 | 1 | 1 | 8 |
|  | All | 1 | 1 | 1 | 11 | 1 | 1 | 1 | 8 | 1 | 1 | 4 | 104 |
|  | | | | | | | | | | | | | |
| Referral | White | 8 | 11 | 13 | 18 | 8 | 11 | 13 | 18 | 8 | 11 | 14 | 21 |
|  | Black | 7 | 10 | 13 | 20 | 7 | 10.5 | 13 | 18 | 7 | 9.5 | 14 | 45 |
|  | Asian | 8 | 11 | 14 | 19 | 8 | 11 | 14 | 17 | 7 | 11 | 14 | 28 |
|  | Mixed | 8 | 11 | 14 | 20 | 8 | 11 | 14 | 20 | 6 | 10 | 13 | 14 |
|  | Other | 8 | 10 | 13 | 18 | 8 | 10 | 13 | 16 | 6 | 8 | 15 | 19 |
|  | All | 8 | 11 | 13 | 19 | 8 | 11 | 13 | 18 | 8 | 11 | 14 | 22 |
|  | | | | | | | | | | | | | |
| Secondary care | White | 7 | 11 | 19 | 69 | 7 | 11 | 18 | 60 | 8 | 14 | 33 | 208 |
|  | Black | 8 | 13 | 26 | 91 | 8 | 13 | 26 | 62 | 7 | 9.5 | 36 | 156 |
|  | Asian | 7 | 12 | 20 | 82 | 7 | 11 | 19 | 74 | 11 | 18 | 48 | 117 |
|  | Mixed | 7 | 11 | 18.5 | 107 | 7 | 11 | 17 | 76 | 8 | 15 | 45 | 197 |
|  | Other | 7 | 10 | 21 | 71 | 7 | 10 | 19 | 56 | 13 | 24 | 59 | 322 |
|  | All | 7 | 11 | 19 | 72 | 7 | 11 | 18 | 61 | 8 | 15 | 34 | 207 |

p25, p50, p75, p95 = 25^th^, 50^th^, 75^th^ and 95^th^ percentile, respectively

**S3: Crude and adjusted odds ratio of ethnic differences in diagnostic intervals by symptoms type - lump vs non-lump symptoms.**

| **Intervals** | **CTR** | **ATR** | **95% CI** | **P-value** |
| --- | --- | --- | --- | --- |
| Diagnostic | **1.58** | **1.56** | **1.31-1.86** | **<0.0001** |
| Primary care | **1.43** | **1.49** | **1.16-1.92** | **0.002** |
| Referral | 1.05 | 1.05 | 0.97-1.13 | 0.23 |
| Secondary care | **1.43** | **1.41** | **1.14-1.74** | **0.002** |

*CTR=crude time ratios, ATR=adjusted time ratios. The analysis was adjusted for age, deprivation, comorbidity, and year of diagnosis (before vs. during the lockdown).*

**S4: Association between ethnicity and diagnostic intervals, including sensitivity analyses excluding cases diagnosed from March 2020 onward to account for potential COVID-19 lockdown effects on diagnostic intervals.**

| **Diagnostic interval** | | | | | | | | |
| --- | --- | --- | --- | --- | --- | --- | --- | --- |
| **Ethnicity** | **Total** | **Model 3** | | | N | **Excluding lockdown cases** | | |
|  |  | **ATR** | **95% CI** | **P-value** |  | **ATR** | **95% CI** | **P-value** |
| White | 7390 |  | | | 6290 |  | | |
| Black | 228 | **1.38** | **1.11-1.71** | **0.003** | 199 | **1.36** | **1.09-1.71** | **0.007** |
| Asian | 366 | 0.97 | 0.85-1.11 | 0.69 | 311 | 1.02 | 0.88-1.17 | 0.83 |
| Mixed | 464 | 0.99 | 0.87-1.13 | 0.86 | 381 | 1.02 | 0.90-1.17 | 0.73 |
| Other | 174 | 0.93 | 0.73-1.19 | 0.57 | 148 | 0.88 | 0.71-1.08 | 0.22 |
| **Total** | 8622 |  | | | 7329 |  |  |  |
| **Primary care interval** | | | | | | | | |
| White | 7390 |  | | | 6290 |  | | |
| Black | 228 | 1.19 | 0.91-1.54 | 0.20 | 199 | 1.19 | 0.93-1.53 | 0.16 |
| Asian | 366 | **0.88** | **0.81-0.96** | **0.004** | 311 | **0.89** | **0.82-0.97** | **0.01** |
| Mixed | 464 | 0.96 | 0.87-1.08 | 0.52 | 381 | 0.96 | 0.86-1.07 | 0.47 |
| Other | 174 | 0.88 | 0.73-1.06 | 0.19 | 148 | 0.90 | 0.74-1.09 | 0.29 |
| **Total** | **8622** |  | | | **7329** |  |  |  |
| **Referral interval** | | | | | | | | |
| White | 7390 |  | | | 6290 |  | | |
| Black | 228 | 0.98 | 0.83-1.16 | 0.80 | 199 | 0.95 | 0.79-1.14 | 0.55 |
| Asian | 366 | 0.98 | 0.88-1.09 | 0.69 | 311 | 0.96 | 0.87-1.07 | 0.49 |
| Mixed | 464 | 0.99 | 0.87-1.11 | 0.81 | 381 | 1.02 | 0.91-1.14 | 0.76 |
| Other | 174 | 0.95 | 0.76-1.19 | 0.67 | 148 | 0.92 | 0.73-1.17 | 0.51 |
| **Total** | **8622** |  | | | **7329** |  |  |  |
| **Secondary care interval** | | | | | | | | |
| White | 7390 |  | | | 6290 |  | | |
| Black | 228 | **1.45** | **1.05-2.01** | **0.02** | 199 | **1.39** | **0.98-1.97** | **0.06** |
| Asian | 366 | 1.05 | 0.84-1.31 | 0.69 | 311 | 1.14 | 0.90-1.44 | 0.28 |
| Mixed | 464 | 0.95 | 0.75-1.21 | 0.68 | 381 | 0.98 | 0.76-1.27 | 0.90 |
| Other | 174 | 1.02 | 0.68-1.53 | 0.94 | 148 | 0.92 | 0.64-1.33 | 0.67 |
| **Total** | 8622 |  | | | 7329 |  | | |
